# Evaluation of the Degree of Polymerization of the Proanthocyanidins in Cranberry by Molecular Sieving and Characterization of the Low Molecular Weight Fractions by UHPLC-Orbitrap Mass Spectrometry

**DOI:** 10.3390/molecules24081504

**Published:** 2019-04-17

**Authors:** Claudio Gardana, Paolo Simonetti

**Affiliations:** DeFENS—Department of Food, Environmental and Nutritional Sciences, Università degli Studi di Milano, Via Celoria 2, 20133 Milano, Italy; paolo.simonetti@unimi.it

**Keywords:** *Vaccinium macrocarpon*, proanthocyanidins, DMAC assay, UHPLC-DAD-Orbitrap MS, molecular sieve

## Abstract

4-dimethylammino-cinnamaldehyde (DMAC) assays quantify total proanthocyanidins (PACs) but do not provide qualitative PAC molecular weight distribution information and cannot discriminate between A- and B-type PACs. We developed an efficient method for assessing PAC molecular weight distributions. The PACs from three commercial cranberry extracts (A1–A3) were fractionated by molecular sieves with cut-offs of 3, 10, 30, 50, and 100 kDa, and each fraction was analyzed by DMAC assays. A1, A2, and A3 contained 27%, 33%, and 15% PACs, respectively. Approximately 28 PACs, 20 flavonols, and 15 phenolic acids were identified by UHPLC-DAD-Orbitrap MS in A1 and A3, while A2 contained only flavan-3-ols. Epicatechin was the main monomer in A1 and A3, and catechin was the main in A2. Procyanidin A2 was the main dimer in A1 and A3, representing more than 85% of the total dimers, while it constituted approximately only 24% of A2. A1 and A3 contained quercetin, isorhamnetin, myricetin, and their glycosides, which were totally absent in A2. In A1 and A3 the PACs were mainly distributed in the fractions 30–3 and <3 kDa, while in A2 more than 70% were present in the fraction less than 3 kDa. Overall, obtained data strongly suggests that A2 is not cranberry-derived, or is adulterated with another source of PACs.

## 1. Introduction

Cranberry, *Vaccinium macrocarpon*, has various biological benefits for human health including the prevention of microbial adhesion in urinary tract infections (UTIs) [1], reduction in biofilm formation [2], antioxidant action [3], cholesterol reduction [4], and anticancer effects [5]. In particular, UTIs are very common and are responsible for approximately 10 million doctor visits annually in the USA [6], and it has been estimated that about 30% of women diagnosed with a UTI will suffer a recurrence within six months [7].

Several mechanisms have been proposed for the actions of cranberry in the prevention of UTIs, with attention especially on its interference with bacterial adhesion in the urinary tract [1]. 

Cranberry has a complex phytochemical composition including mainly flavon-3-ols, anthocyanins, aromatic acid, and monomeric flavan-3-ols together with oligomeric and polymeric proanthocyanidins (PACs), respectively [8]. Flavanones and stilbenes has been found in cranberries in lower amounts [9].

Cranberry flavon-3-ols occur mainly as glycosylated forms of quercetin, myricetin, and kaempferol, respectively [10], and their total amount in the fruit is in the range of 0.3–0.5 mg/kg [11]. 

Regarding anthocyanins, cranberry seems to have a unique qualitative profile [12]. Indeed, fruit contains four main anthocyanins corresponding to peonidin-galactoside, peonidin-arabinoside, cyanidin-arabinoside, and cyanidin-galactoside. Peonidin-glucoside and cyanidin-glucoside have been found in lower amounts. The monomeric anthocyanin content ranged from 25 to 70 mg/100 g FW, and galactosides, arabinosides, and glucosides comprised approximately 53, 42, and 5% of the total anthocyanins, respectively [12,13,14]. 

Among these phytochemicals, anti-UTI action has been attributed to the proanthocyanidin fraction [15]. The oligomeric and polymeric nature of PACs has several structural variations depending on the degree of polymerization (DP), linkage types [i.e., C-C bond (B-type) or a CC- and ether bond (A-type)], interflavan bond positions (describe C-C positions), and type of monomeric units. Catechin and epicatechin are the two most common flavan-3-ol units present in PACs. Gallocatechin and epigallocatechin units, which present an additional hydroxyl group, are also present [16]. Since these four monomers and the several different linkages can be distributed randomly within the polymer, the number of possible isomers of PACs increases exponentially with the degree of polymerization. For example, for a 20-unit degree of polymerization, approximately five hundred thousand PACs are theoretically possible [16]. In addition, at DP greater than 2, both A- and B-types may be present. Because of these issues, and the lack of reference standards, PAC quantification has been problematic. Thiolysis has been used to estimate the average DP of cranberry. DPs of 4.7 and 8.5 have been reported [17,18] and subsequently, by MALDI-TOF MS, a DP higher than 23 has been detected [16]. Establishing the DP of PACs in cranberry is problematic because what is detected in a given sample depends on several factors, such as the origins of the plant materials and methods of preparation used.

Due to the complexity of PACs in terms of the large ranges of molecular weights and linkage types, currently there is no universally accepted standard method for their quantification. The methods currently in use to quantify total PACs include those based on hydrolysis in an acidic solution with the formation of colored compounds (Bate-Smith), and on hydrolysis with the production of monomers that are determined by HPLC (thiolysis and phloroglucinolysis), gravimetric, and colorimetric (vanillin or 4-dimethylamino-cinnamaldehyde (DMAC)) analyses. Among these, the most used are the Bate-Smith, vanillin acid, and DMAC assays. In the Bate-Smith assay, PACs are hydrolyzed in n-butanol-HCl to produce anthocyanidins, which are then quantified spectrophotometrically at 520–550 nm [19]. The Bate-Smith assay has drawbacks, such as possible incomplete PAC hydrolysis and transformation, and the lack of a suitable extinction percentage coefficient.

In the vanillin acid assay, the aldehyde group reacts with PACs, forming a colored derivative that absorbs at 510 nm. The presence of anthocyanins may confound the measured absorbance, and compounds such as ascorbic acid and other flavonoids may lead to overestimation of the PAC amount [20,21].

Regarding the DMAC assay, in acidic solutions, the reagent gives a strongly reactive electrophilic carbocation that reacts selectively with compounds with meta-oriented di- or tri-hydroxyl phenols, as found in PACs. The reaction produces a green derivative that absorbs at 640 nm. DMAC does not react with hydroxyl-phenylalkyl acids, ascorbic acid, or other flavonoids. Thus, it seems more specific and reproducible than the Bate-Smith and vanillin assays. The belief that the molar absorption coefficient is constant across the various PAC species is the most significant reason why the DMAC assay is preferred to this day [22]. However, Feliciano et al. showed that the standard used in the DMAC assay, procyanidin A2, leads to an underestimation of the PAC content in cranberry products, especially those containing higher molecular weight PACs [23]. Indeed, the slope of the PAC standard curve was 2.5 times lower than those of procyanidins A2 and B2 were, and it was 7.1 times lower than that of catechin, indicating that the PAC content in cranberry would be underestimated by 2.5- or 7-fold if these standards were used for the DMAC assay. 

The reported methods give quantitative information, but they do not provide qualitative information on PAC molecular weight distributions, nor can they discriminate between A- and B-type PACs.

To get qualitative information, PACs have been analyzed by MALDI-TOF [16,24] or liquid chromatography coupled to low- [25,26,27,28] or high-resolution mass spectrometers [29,30,31]. These techniques allow the determination of both the low molecular weight PACs (DP < 10) and the distinction between type A and B. All too often, though, the PACs must be isolated from the matrix before analysis, and this requires lengthy and time-consuming methods such as those that need open-column chromatography [32,33]. 

The purpose of our study was to develop a simple method for fractioning PACs from cranberry extracts. Thus, the PACs of three different commercial extracts were split by ultra-centrifugal filter with cut-offs of 3, 10, 30, 50, and 100 kDa, and each fraction was analyzed by the DMAC assay to assess total PACs. Finally, the fractions lower than 3 KDa were also analyzed by UHPLC-DAD-Orbitrap MS to determine monomers and oligomers.

## 2. Results

### 2.1. Total Amount of PACs in Commercial Extracts

The use of procyanidin A2 as a standard for the DMAC assay has been shown to underestimate total PACs content compared to cranberry-derived PACs. Nevertheless, in this study the evaluation of total PACs was carried out using PA2 for both because it is normally used in quality control laboratories.

The cranberry extracts were entirely soluble in the extraction solution. The total amounts of PACs in A1, A2, and A3, as determined by the DMAC assay, were 27.1 ± 1.1, 33.1 ± 2.1, and 14.7 ± 1.0% (Table 1), respectively. The repeatability and inter-day precision were in the range of 3.9–6.3% and 4.3–7.3%, respectively. These results are in good agreement with those reported by Prior et al. [34]. 

### 2.2. PACs Determination by UPLC-DAD-Orbitrap MS

**Figure 1:**
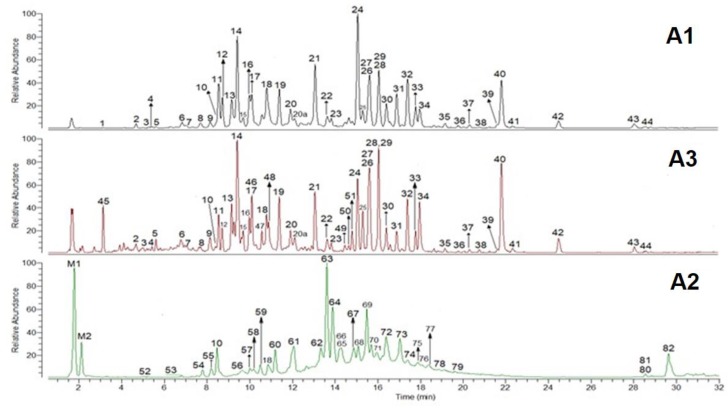
The total ion chromatography, 100–2000 u, of cranberry extracts A1, A2, and A3. See Table 2 for peak identifications.

Low molecular weight flavan-3-ols in the cranberry extracts have been characterized by reversed phase UHPLC coupled to DAD and Fourier transform mass spectrometers operating in the negative mode. High mass resolution (50 K) and high mass accuracy (2 ppm) allow the empirical formula of deprotonated and fragmented ions to be obtained. These features, together with enhanced efficiency of the UHPLC technique, made the system we used a powerful tool for the identification of unknown analytes in the cranberry extract. Untargeted analysis, however, cannot be done based only on elemental composition data. Additional information is required, such as UV spectra and fragmentation patterns with CIDs of the parent ions. An example of the UHPLC-HR MS profiles of sample A1, A2, and A3, extracted in the range of 100–2000 u, is shown in Figure 1. Table 2 reports the on-line UV spectra, deprotonated ion, and fragments of the main compounds such as flavan-3-ols and flavon-3-ols detected in cranberry extracts. 

The main monomers found in all the extracts were EC and CAT. Epicatechin was the main monomer in A1 and A3, representing more than 90% of the total monomers (Table 1). However, CAT was the main monomer in A2, and it accounted for more than 80% of the total monomers. These data are in agreement with some authors’, which reported that in cranberry, epicatechin was more abundant than catechin [28]. Regarding dimers, the A-types were the main dimers in all of the extracts, representing approximately more than 80% of the total dimers in A1 and A2 and 60% in A3 (Table 1). Procyanidin A2 was the main dimer in A1 and A3, representing more than 75% and 60% of the total dimers, respectively. Regarding A2, PA2 was not the main dimer; it constituted approximately 5% of the total dimers. The main dimer in A2 was the peak 63 (Figure 1), retention time (RT) 13.5 min, which constituted approximately 55% of the total dimers. 

AB-types were the main trimers in A1, A2, and A3 and represented approximately 72%, 90%, and 76% of the total trimers, respectively. AA-types were not found in A3, while in A1 and A2 they represented approximately 7% of the total trimers. Proanthocyanidin C1 was the main BB-type trimer in A1 and A3, representing approximately 60% of the total BB-types. On the contrary, PC1 was not detected in the extract A2. The main BB-type trimer in A2 was the peak 52, RT 5 min (Figure 1), which constituted approximately 57% of the trimers BB-type. 

Catechin and EC showed the same fragmentation pattern, and the most abundant ion had an *m/z* of 123.0454 u, corresponding to a 3,4-dihydroxy-toluene moiety (B-ring). Dimers of A- and B-type gave a different fragmentation pattern, and the main ions had *m/z* of 285.0410 (C_15_H_9_O_6_) and 289.0732 u, respectively. Besides the deprotonated ions, the MS spectra of the A- and B-type dimers showed the presence of the dimer [2M-H]^-^. Adducts with formic acid or doubly charged ions were not detected. 

Four main trimers, with an *m/z* of 863.1830 u, containing one A-type bond, were present in the extract A1 and A2. Three of them had a common fragmentation pattern, and the main ions had *m/z* of 575.1220, 693.1280, 449.0900, 285.0419, and 711.1388 u. The other AB-trimer, RT 11.9 min, produced mainly ions with *m/z* of 411.0726 and 289.0721 u. Ions with *m/z* of 1727.3725 and 1731.4040 u, which corresponded to the dimer [2M − H]^-^, were present in much lower quantities. Adducts or doubly charged ions were not detected. Two main BB-type trimers, with *m/z* of 865.1990 u, were present in the extracts A1 and A3, and one of them was PC1, RT 12.1 min. The other trimer had lower retention time, 10 min, indicating that one of the monomers or both could be catechin. These trimers shared the fragment with *m/z* of 407.0791, 287.0574, 577.1368, and 125.0247 u. In addition, the trimer with RT 10 min also showed a fragment with *m/z* of 160.0169 u, which matched the formula C_9_H_4_O_4_. The MS spectra of a compound with DP > 3 showed the presence of doubly charged ions [M − 2H]^2-^. In particular, for the ABB-type tetramer detected in the extract A2, Peak 57 (Figure 1), the doubly charged ions were the most abundant species.

Signals attributable to glycosylated-flavonols and their aglycones were almost exclusively detected at 354 and 370 nm, respectively. Thus, twenty peaks have been tentatively identified, most of which were glycosylated forms of quercetin and myricetin, present with the corresponding aglycones. Minor signals were assigned to glycosides of isorhamnetin and methyl-myricetin.

Regarding the non-flavanol fraction, the A1 and A3 samples differed significantly from A2. Indeed, A1 and A3 contained phenolic acid and flavonol derivatives, which were totally absent in A2. In particular, they contained glycosylated forms of quercetin, isorhamnetin, and myricetin. Quercetin was the main aglycone, while the major glycosides were quercetin-3-*O*- and myricetin-3-O-glucoside. The identities of these compounds was then confirmed by an authentic standard. These data were in agreement with those reported by different authors [35]. 

Identification of some phenolic acids by MS could be difficult due to the presence of the isobaric moieties glucose (C_6_H_12_O_6_) and caffeic acid (C_9_H_8_O_4_), which both present an *m/z* ratio of 179 u. Differing by 118 ppm, they are indistinguishable with a low resolution mass spectrometer but can be easily resolved by the high resolution MS used in this study. Thus, several phenolic acids have been detected and identified (Table 2), and the main ones were glycosides of caffeic and *p*-coumaric acid. Moreover, glycosylated forms of benzoic, vanillic, sinapic, 4-hydroxy-benzoic, and 3,4-dihydroxy-benzoic acid were found in lower amounts.

### 2.3. Proanthocyanidin Fractioning by Molecular Sieve

Regarding the reaction of the DMAC with flavanols, several authors report that the maximum absorption at 640 nm occurs after about 20 min [34], while others report shorter times, about 12 min [36]. Accordingly, even considering that fractions with different molecular weights could react with DMAC faster or slower, the reaction of the flavan-3-ols with DMAC was monitored every minute for 60 min to evaluate maximum absorption.

**Figure 2:**
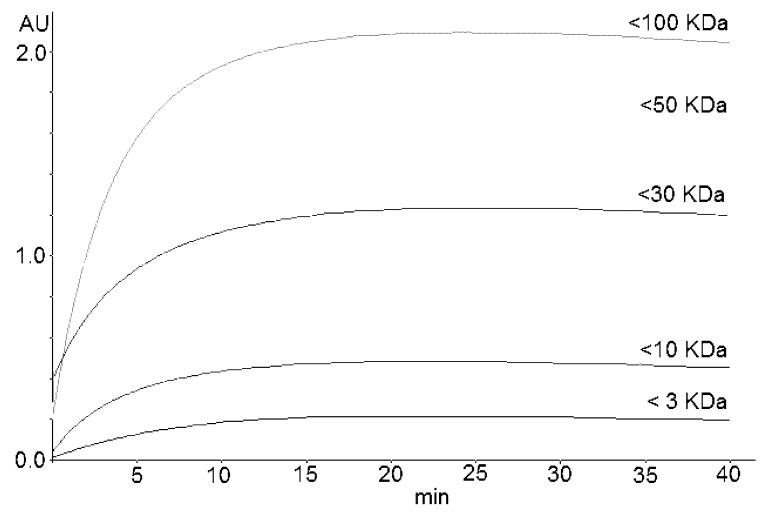
Kinetics of the DMAC reaction of permeates obtained by fractionating the cranberry extract A3. Permeates were obtained by molecular sieves with cut-offs of 3, 10, 30, 50, and 100 kDa.

The reaction stabilizes to a plateau in the ranges of 18–25, 15–20, and 20–30 min for A1, A2, and A3, respectively. Figure 2 shows the kinetics obtained by reacting the permeates (obtained by fractionating A3 using molecular sieves) with DMAC. Table 3 reports the percentage distribution of PACs in the commercial cranberry extracts that were analyzed. 

In all samples, approximately 90% of PACs had molecular weights less than 30 kDa, and there were some differences between the extracts. In particular, in A1 and A3, the PACs were mainly distributed in the fractions 30–10, 10–3 and < 3 kDa, while in A2, more than 70% were present in the low molecular weight fraction, which was less than 3 kDa. In this regard, it must be emphasized that the solution behavior of PACs is subject to aggregation due to hydrogen bonding among PACs and with other molecules such as phenolics and carbohydrates. Thus, the molecular weight cut-offs of the used filters may not be an accurate indicator of the PAC molecular weight.

The composition of the analyzed extracts showed significant variations in terms of the relative amounts of different PACs as well as flavonols. Extract A2 differed from A1 and A3 with regard to the main monomer, CAT, rather than EC, and the main dimer. The lack of well-established cranberry constituents strongly suggests that A2 is not cranberry-derived, or is adulterated with another source of PACs [37]. 

Thus, the standardization of cranberry extract should be carried out both by spectrophotometric assay and LC-MS analysis, leading to the determination of PACs and other flavonoids. Cranberry extracts used in clinical studies were poorly standardized, and this led to conflicting results and made it difficult to compare the outcomes. Our “multi-component” standardization could facilitate the interpretation of results from different clinical studies.

Overall, the procedure developed to obtain fractions containing PACs with different molecular weights is reproducible and faster than those using open columns containing resins such as Sephadex LH-20 and subsequent chromatography with preparative HPLC [38]. Furthermore, the availability of molecular sieves with different cut-offs from those we used will allow others to obtain further fractions to better characterize the PACs of cranberry.

## 3. Materials and Methods 

### 3.1. Chemicals and Materials

Standards of catechin (CAT), epicatechin (EC), myricetin-3-*O*-glucoside, quercetin-3-*O*-glucoside, quercetin, procyanidin C1 (PC1), and procyanidin A2 (PA2) were purchased from Extrasynthese (Genay, France). Methanol, acetonitrile, 4-dimethylammino-cinnamaldehyde (DMAC), and acetic acid were from Sigma-Aldrich (St. Louis, MO, USA). Amicon ultra-4 centrifugal filter units of 3, 10, 30, 50, and 100 nominal molecular weight limits (NMWL) were supplied from Merck Millipore (Milan, Italy). Water was from a Milli-Q apparatus (Millipore, Milford, CT, USA). Commercial dried cranberry extracts (A1, A2, and A3) were obtained from different manufacturers. Notably, A1 and A3 were obtained from an industrial producer of natural ingredients starting from whole berry. On the contrary, A2 was produced by a small company through a proprietary purification and concentration process starting from cranberry commercial extracts. Details regarding the plant origins and manufacturing processes of the extracts are not available.

### 3.2. Determination of Total Proanthocyanidins

Approximately 25 mg of the cranberry extracts were dissolved in 40 mL of a solution of acetone:water:acetic acid (75:24.5:0.5 *v/v/v*). The mixture was vortexed for 30 sec, sonicated for 10 min, and then the volume was adjusted to 50 mL by a solution of acetone:water:acetic acid (75:24.5:0.5 *v/v/v*). The extract was diluted 2-, 5-, 10-, 20-, and 50-fold for DMAC assays. For the calibration, a stock solution (1 mg/mL) of PA2 was prepared by dissolving 20 mg of the standard in 20 mL of methanol. This solution was subsequently diluted with a solution of acetone:water:acetic acid (75:24.5:0.5 *v/v/v*) to produce six working solutions in the range of 2–50 µg/mL. Total PAC was determined according to Prior et al. [34], with slight variations. Briefly, an acidified ethanol solution was prepared by adding 12.5 mL of 37% HCl to 12.5 mL of deionized water and 75 mL of ethanol, and then 50 mg of DMAC reagent was dissolved in 50 mL of this solution immediately prior to use. Then 70 µL of sample or standard was added to 2.1 mL of DMAC solution, and the reaction was monitored at 640 nm every minute for 60 min by a Lambda 20 spectrophotometer (PerkinElmer, Waltham, MA, USA). The blanks were reagents and samples diluted in acidified ethanol. The assay was performed in triplicate, and the total percentages of the PACs, expressed as PA2 equivalents, were calculated as follows:

$$\mathrm{Total}\;\mathrm{PACs}\;\left(\%\right)=\;\frac{\left(A-q\right)VD}{10m\;W}$$

where *A* is the absorbance (AU), *q* is the intercept of the procyanidin A2 calibration curve (0.004), *m* is the slope of the PA2 calibration curve (0.024), *V* is the extraction volume (mL), *D* is the dilution factor, and *W* is the sample weight (mg).

### 3.3. Proanthocyanidin Determination by UHPLC-DAD-Orbitrap MS 

Approximately 20 mg of cranberry extract were dissolved into 5 ml of a methanol:water (60:40, *v/v*) solution. The mixture was centrifuged at 500× *g* for 10 min, and the supernatant transferred to a 10 mL volumetric flask. The residue was washed with 4 mL of a methanol:water (60:40, *v/v*) solution, and the mixture was treated as described above. The resulting solutions were mixed, and water was added to adjust the volume. The solution was centrifuged at 1000× *g* for 2 min, and 5 µL was injected into the UHPLC system. The analysis was performed on an Acquity UHPLC system (Waters, Milford, MA, USA) coupled with an eLambda DAD (Waters) and a high-resolution Fourier Transform Orbitrap mass spectrometer, Exactive model (Thermo Scientific, Rodano, Italy), equipped with a HESI-II probe for ESI and a collision cell (HCD). The operative conditions were as follows: Spray voltage −3.0 kV, sheath gas flow rate 55 (arbitrary units), auxiliary gas flow rate 20 (arbitrary units), capillary temperature 350 °C, capillary voltage −60 V, tube lens −100 V, skimmer −26 V, and heater temperature 130 °C. A BEH Shield C_18_ column (150 × 2.1 mm, 1.7 µm, Waters) maintained at 50 °C was used for the separation. The flow rate was 0.45 mL/min, and the eluents were 0.05% formic acid in water (A) and acetonitrile (B). The UHPLC separation was achieved by the following linear elution gradient: 5–35% of B for 10 min, which was then increased from 35–80% B for 10 min. The acquisition was made in the full-scan mode in the range (*m/z*)^-^ 100–2000 and 200–4000 u, using an isolation window of ±2 ppm. The AGC target, injection time, mass resolution, energy, and gas in the collision cell were 1 × 10^6^, 100 ms, 50 K, 60 V, and N_2_, respectively. The MS data were processed using Xcalibur software (Thermo Scientific). The peak identity was ascertained by evaluating the accurate mass, the fragments obtained in the collision cell, and the on-line UV spectra (200–450 nm).

Catechin, EC, PC1, and PA2 stock solutions (1 mg mL^-1^) were prepared in methanol and stored at −20 °C. Working solutions (*n* = 5) were prepared in the range of 0.2–20 µg/mL and stored at 4 °C. Analysis was carried out in duplicate. The amounts of the dimers and trimers that were not available as reference standard were estimated using the PA2 and PC1 calibration curve equations, respectively. 

### 3.4. Proanthocyanidin Fractioning by Ultra-Centrifugal Filter

Approximately 100 mg of the cranberry extract was dissolved in 40 mL of a methanol:water (50:50, *v/v*) solution. The mixture was vortexed for 1 min, sonicated for 10 min, and then the volume of the clear solution was adjusted to 50 mL by a methanol:water (50:50, *v/v*) solution. The solutions were diluted and analyzed by DMAC assays to determine total PACs.

**Figure 3:**
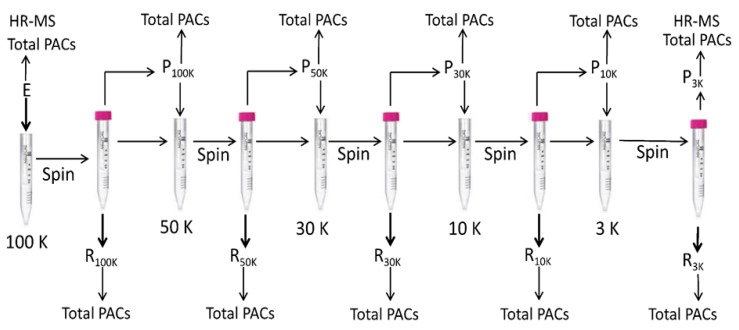
Fractionation of the proanthocyanidins (PACs) by centrifugal filter devices. The extract solution was centrifuged at 4000× g by a centrifugal filter device containing an ultracel-PL PLHK 100 kDa regenerated cellulose membrane, which allowed the ultrafiltration into the lower chamber (permeate) of substances with MW  < 100 kDa. Substances with MW > 100 kDa were collected in the upper chamber (retained). Permeate was then loaded on a 50 kDa filter and centrifuged at 4000 × *g* until the solution passed completely through the filter. The procedure was repeated on 30, 10, and 3 kDa filters. Permeates and retained fractions were analyzed by the DMAC assay, and the permeates less than 3 kDa were analyzed by UHPLC-DAD-HR MS.

Then 4 mL of the extract was loaded on a 100K NMWL filter, which was then centrifuged at 4000 × *g* until the solution was completely passed through the filter. Permeate was transferred to a 10 mL tube, and the volume was adjusted by methanol. The residue (retained) was dissolved in 4 mL of a methanol:water (50:50, *v/v*) solution, transferred to a 5 mL flask, and the volume was adjusted with methanol. Permeate (4 mL) was loaded on a 50K NMWL filter and treated as described above. The procedure was then repeated on 30, 10, and 3 K NMWL filters. Permeate and residue were analyzed by DMAC assay to determine total PACs and the 3K NMWL permeate was also analyzed by UHPLC-DAD-MS. The entire procedure is schematized in Figure 3.

### 3.5. Statistical Analysis

The statistical analysis was performed using Excel software. The singly charged ions of the different PACs were calculated according to the following equation:

HRMW = 12(15DP) + 1.0078(12DP + 2 – 2A) + 15.9949(6DP) – 1.0078

where DP was the polymerization degree, and *A* was the number of A-type bonds. The masses of C, H, and O were 12, 1.0078, and 15.9949, respectively.

## Figures and Tables

**Table 1:** Total PACs in Cranberry extracts by DMAC assay and percentage of the main monomers, dimers, and trimers quantified by UHPLC-HR MS.

Analyte	A1 (%)	A2 (%)	A3 (%)
Total PACs	27.1 ± 1.1^a^	33.1 ± 2.1^a^	14.7 ± 1.0^a^
Catechin	0.1 ± 0.0	0.4 ± 0.0	0.0 ± 0.0
Epicatechin	1.3 ± 0.1	0.1 ± 0.0	0.4 ± 0.0
Dimers, A-type	1.8 ± 0.1	3.3 ± 0.1	0.5 ± 0.0
(PA2)	1.8 ± 0.1	0.2 ± 0.0	0.5 ± 0.0
Dimers, B-type	0.5 ± 0.0	0.5 ± 0.1	0.3 ± 0.1
Trimers, AA-type	0.1 ± 0.0	0.2 ± 0.1	n.f.
Trimers, AB-type	1.4 ± 0.1	2.5 ± 0.1	0.5 ± 0.0
Trimers, BB-type	0.4 ± 0.0	0.1 ± 0.0	0.1 ± 0.0
(PC1)	0.2 ± 0.0	n.f.	0.1 ± 0.0

^a^ DMAC assay, n.f. not found (<LOD).

**Table 2:** Compounds identified in the analyzed commercial cranberry extracts (A1-A3). For each compound is reported the retention time (RT, min), λ_max_ (nm), HR mass of the deprotonated ion [M-H]^-^, molecular formula, and fragment ions [M − H]^−^.

Peak	RT	λ_max_	[M − H]^−^	Formula	Fragment Ions	Compound
1	3.2	270	169.0146	C7 H5 O5	125.0246	GA
2	4.7	315	315.0735	C13 H15 O9	152.0118	DHBA-Hex
3	5.3	277	329.0883	C14 H17 O9	167.0353, 152.0118, 123.0454	VA-Hex
4	5.4	263	299.0784	C13 H15 O8	137.0247	HBA-Hex
5	5.6	302	315.1091	C14 H19 O8	163.0404, 153.056, 145.0298, 123.0454	VH-Hex
6	6.8	295	503.1428	C21 H27 O14	341.0883, 179.0354, 161.0249, 135.0455	CA-di-Hex
7	7.0	328	341.0884	C15 H17 O9	179.0354, 161.0249, 133.0298	CA-Hex
8	7.7	277	577.1363	C30 H25 O12	407.0792, 289.0731, 125.0247	DP2, B
9	8.1	310	341.0884	C15 H17 O9	179.0354, 161.0249, 133.0298	CA-Hex
10	8.4	227, 277	289.0726	C15 H13 O6	159.0456, 137.0247, 123.0455	CAT
11	8.5	320	353.0884	C16 H17 O9	191.0567, 163.0567	CHL
12	8.7	313	325.0938	C15 H17 O8	163.0404, 145.0298	pC-Hex
13	9.1	234, 277	591.1147	C30 H23 O13	447.0947, 347.0576, 284.0339	DP2 A^1^
14	9.4	234	461.1307	C19 H25 O13	121.0297	HBA-di-Hex
15	9.7	241	445.1360	C19 H25 O12	323.0989, 121.0297	BA-di-Hex
16	10.0	234, 277	865.2005	C45 H37 O18	407.0791, 287.0574, 577.1368 160.0170, 125.0247	DP3, BB
17	10.1	234	461.1307	C19 H25 O13	121.0297	HBA-di-Hex
18	10.9	227, 277	289.0726	C15 H13 O6	159.0456, 137.0247, 123.0455	EC
19	11.4	234	371.0991	C16 H19 O10	359.1515, 344.1277, 249.0625, 121.0298	BA-X
20	11.9	234, 278	863.1857	C45 H35 O18	573.1061, 451.1053, 411.0742, 289.0732	DP3, AB
20a	12.1	234, 280	865.2007	C45 H37 O18	407.0791, 287.0574, 577.1368, 125.0247	PC1
21	13.1	255, 356	479.0841 959.1754	C22 H19 O13 [2M-H]^-^	316.0237, 271.026	M-Glc
22	13.6	230, 280	863.1853	C45 H35 O18	575.1894, 449.1099	DP3, AB
23	13.8	230, 275	577.2055	C32 H33 O10	397.1423, 373.1157, 203.0833	N.I.
23a	14.8	254, 311	535.1469	C25 H27 O13	316.0238, 271.0262, 191.0356, 163.0405, 147.0455	N.I.
24	15.0	255, 356	463.0893 927.1860	C21 H19 O12 [2M-H]^-^	300.0290, 271.0261, 151.0040	Q-Glc
25	15.3	254, 312	535.1469	C25 H27 O13	407.0789, 289.0730, 191,0356, 163.0405, 147.0455	N.I.
26	15.5		493.1008	C22 H21 O13	330.0395, 315.0161, 163.0404	methyl-M
27	15.6	230, 280	575.1200 1151.2485	C30 H23 O12 [2M-H]^-^	285.0417	PA2
28	16.0	313	537.1624 1075.3317	C25 H19 O13 [2M-H]^-^	163.0405, 149.0611	dh-MT-pC
29	16.1		433.0783	C20 H17 O11	300.0289, 271.0261	Q-Ara
30	16.4	255, 352	433.0787	C20 H17 O11	300.0288, 271.0260, 255.0310, 151.0040	Q-Ara
31	16.9	255, 352	433.0787	C20 H17 O11	300.0288, 271.0260, 255.0310	Q-Ara
32	17.4	255, 348	447.0943	C21 H19 O11	300.0288, 271.0259, 255.0310, 151.0040	Q-Rha
33	17.8	252, 359	507.1155	C23 H23 O13	344.0551, 329.0319, 316.0601, 301.0367, 273.0419	dm-M-Hex
34	17.9	252, 377	317.031	C15 H19 O8	271.0259, 151.004	M
35	19.2	254, 352	447.0951	C21 H19 O11	314.0447, 285.0419, 271.0262, 243.0309	I-Ara
36	19.8	252, 348	447.0951	C21 H19 O11	314.0447, 285.0419, 271.0262, 243.0310	I-Ara
37	20.3	262, 359	477.1049	C22 H21 O12	344.0551, 329.0317, 316.0601, 301.0367, 273.0418	dm-M-Pentose
38	20.8	280, 316	445.1158	C22 H21 O10	179.0355, 135.0455, 121.0297	CA-Hex-BA
39	21.6	285, 310	609.1270	C30 H25 O14	463.0902, 300.0288, 163.0041, 151.0040	Q-Hex-pC
40	21.8	255, 370	301.0363	C15 H9 O7	151.0040	Q
41	22.3	254, 373	331.0468	C16 H11 O8	316.0239, 271.0262, 164.0120, 151.0041	I
42	24.5	255, 352	567.1158	C28 H23 O13	300.0289	Q-Hex-BA
43	28.1	255, 366	345.0622	C217 H13 O8	315.0519, 300.0288, 287.0211, 271.0260, 151.0041	dm-M
44	28.8	266, 252	593.1318	C30 H25 O13	300.0288, 271.0261	Q-Rha-pC
45	3.2	235	391.1254 783.2287	C16 H23 O11 [2M-H]^-^	229.0724 (A), 211.0618, 167.0717, 149.0611, 123.0454	A-Hex
46	10.1	230, 277	415.1257 461.1311	C18 H23 O11 [M+COOH]^-^	284.0340, 121.0298	N.I.
47	10.5	248, 352	385.1151	C17 H21 O10	205.0513, 190.0287, 175.0042, 149.0248	SA-Hex
48	10.9		385.1877 431.1931	C19 H29 O8 [M+COOH]^-^	298.0495, 283.0260, 205.1240, 153.0925, 125.0247	N.I.
49	14.4	260, 359	449.0737	C20 H17 O12	316.0238, 287.0211, 271.0262	M-Pentose
50	14.6	266, 348	449.0737	C20 H17 O12	316.0238, 287.0212, 271.0262	M-Pentose
51	14.8	248, 309	535.1471 1071.3013	C25 H27 O13 [2M-H]^-^	316.0236, 271.0264, 191.0356, 163.0405, 147.0455	M-Pentose-X
M1	1.8		341.1093 665.2165	C12 H21 O11, C24 H41 O21	161.0456	Maltodextrin
M2	2.1		989.3204 1151.3730 1313.4260	C36 H61 O31 C42 H71 O36 C48 H81 O41	341.1093, 827.2680, 665.2165, 161.0458	Maltodextrin
52	5.0	230, 277	865.2011	C45 H37 O18	407.0791, 287.0574, 577.1368	DP3, BB
53	6.8	230, 277	1439.3153 719.1533	C75 H59 O30 [2M-H]^-^	573.1053, 411.0738, 289.0731, 125.0247	DP5, ABBB
54	7.7	230, 277	577.1367	C30 H25 O12	407.0791, 289.0730, 245.0830, 125.0247	DP2, B
55	8.2	230, 277	577.1367	C30 H25 O12	407.0791, 289.0730, 245.0830, 125.0247	DP2, B
56	9.6	230, 277	577.1367	C30 H25 O12	447.0944, 284.0341, 125.0248	DP2, B
57	10.0	230, 277	1151.2487 575.1208	C60 H47 O24 [M-2H]^2-^	447.0943, 285.0419, 125.0248	DP4, ABB
58	10.2	230, 277	1151.2489 575.1208	C60 H47 O24 [M-2H]^2-^	863.1873, 285.0418, 125.0248	DP4, ABB
59	10.5	230, 277	863.1857	C45 H35 O18	693.1282, 575.1221, 449.0899, 423.0743, 407.0791, 285.0419, 125.0247	DP3, AB
60	11.2	234, 277	863.1857	C45 H35 O18	411.0742, 289.0732	DP3, AB
61	12.1	237, 278	863.1857	C45 H35 O18	693.1282, 575.1221, 449.0899, 289.0732, 125.0247	DP3, AB
62	13.3	232, 278	863.1850	C45 H35 O18	693.1282, 575.1221, 449.0899, 289.0732, 125.0247	DP3, AB
63	13.6	231, 277	575.1221	C30 H23 O12	449.0899, 289.0732, 125.0247	DP2, A
64	13.9	234, 278	863.1850	C45 H35 O18	575.1221, 449.0899, 289.0732, 125.0247	DP3, AB
65	14.2	236, 280	1726.2633 862.1778	C90 H69 O36	1153.2630, 863.1860, 411.0741, 289.0731	DP6, AABBB
66	14.3	234, 278	1437.2966 718.1456	C75 H57 O30 [M-2H]^2-^	1149.2328, 862.1768, 575.1221, 411.0740, 285.0419, 125.0247	DP5, AABB
67	14.9	234, 278	1149.2328	C60 H45 O24	575.1221, 411.0740, 285.0419, 125.0247	DP4, AAB
68	15.1	237, 278	1437.2878	C75 H57 O30	863.1839, 575.1221, 411.074, 285.0419, 125.0247	DP5, AABB
69	15.7	234, 279	575.1212	C30 H23 O12	447.0743. 411.0741, 285.0419, 125.0247	DP2, A
70	15.7	234, 279	575.1212	C30 H23 O12	4479.0902. 411.0745, 285.0419, 125.0247	DP2, A
71	16.0	237, 278	1149.2328	C60 H45 O24	575.1221, 449.0899, 411.0740, 285.0419, 125.0247	DP4, AAB
72	16.4	237, 279	1149.2323	C60 H45 O24	575.1221, 449.0899, 411.0740, 285.0419, 125.0247	DP4, AAB
73	17.0	234, 277	575.1212	C30 H23 O12	285.0419, 125.0247	DP2, A
74	17.4	234, 278	1149.2323	C60 H45 O24	575.1221, 449.0899, 411.0740, 285.0419, 125.0247	DP4, AAB
75	17.9		1149.2323	C60 H45 O24	575.1221, 411.0740, 285.0419, 125.0247	DP4, AAB
76	18.1		1149.2323	C60 H45 O24	861.1715, 573.1060, 411.0740, 125.0247	DP4, AAB
77	18.4		1149.2323	C60 H45 O24	575.1221, 497.2778, 411.0740, 285.0419	DP4, AAB
78	18.9		1149.2323	C60 H45 O24	575.1221, 411.0740, 285.0419, 125.0247	DP4, AAB
79	19.6		1149.2323	C60 H45 O24	575.1219, 411.0740, 285.0419	DP4, AAB
80	28.5		965.4270	C44 H69 O23	803.3750, 641.3204	N.I.
81	28.5		327.2186	C18 H31 O5		N.I.
82	29.6		329.2344	C18 H33 O5	283.0671, 211.1347, 183.1394, 171.1031, 139.1131, 127.1131	N.I.

^1^ dimer EC(C)-EGC, DP: degree of polymerization, N.I.: not identified. Ara: arabinose, BA: benzoic acid, CA: caffeic acid, CAT: catechin, CHL: chlorogenic acid, DHBA: 3,4-dihydroxy-benzoic acid, dh-MT-pC: dihydro-monotropein-p-coumaroyl, dm-M-pentose: di-methyl-myricetin-pentose, dm-M-Hex: di-methyl-myricetin-hexose, EC: epicatechin, GA: gallic acid, Glc: glucose, HBA: hydroxy-benzoic acid, Hex: hexose, I: isorhamnetin, M: myricetin, PA2: procyanidin A2, pC: p-coumaric acid, PC1: procyanidin C1, Q: quercetin, SA: sinapic acid, VA: vanillic acid, VH: vanillyl alcohol, X: unknown residue.

**Table 3:** PAC distribution in commercial cranberry extracts (A1–A3). The relative percentages of PACs were determined by DMAC assays after fractionation by molecular sieves. Values are reported as averages ± S.D.

NMWL	A1	A2	A3
>100 K	0.1 ± 0.0	0.9 ± 0.0	1.1 ± 0.1
100–50 K	2.5 ± 0.1	0.6 ± 0.0	3.8 ± 0.2
50–30 K	4.9 ± 0.2	8.3 ± 0.4	9.2 ± 0.5
30–10 K	21.6 ± 1.1	11.6 ± 0.6	22.4 ± 1.1
10–3 K	36.3 ± 1.4	5.7 ± 0.3	37.8 ± 1.6
<3 K	34.6 ± 1.4	72.9 ± 3.6	25.7 ± 1.2

NMWL: nominal molecular weight limit.
